# Supplementary material for: Resolving the full spectrum of human genome variation using Linked-Reads
Source: Genome Res. 2019 Apr;29(4):635–45. doi: 10.1101/gr.234443.118 (PMC6442396; doi:10.1101/gr.234443.118)
Supplement: Supplemental Material [file supp_gr.234443.118_Supplemental_longranger-2.2.zip › longranger-2.2.tar.bz2!/lr2.2-public/lib/stan/cmdstan/stan/doc/api/html/write__error__msg_8hpp_source.html]

Stan: src/stan/common/write\_error\_msg.hpp Source File


|  |  |
| --- | --- |
|  | Stan  2.5.0  probability, sampling & optimization |


- Main Page
- Related Pages
- Namespaces
- Classes
- Files

- File List
- File Members

All Classes Namespaces Files Functions Variables Typedefs Enumerations Enumerator Friends Macros Pages

- src
- stan
- common

write\_error\_msg.hpp

Go to the documentation of this file.

1 #ifndef STAN\_\_COMMON\_\_WRITE\_ERROR\_MSG\_HPP

2 #define STAN\_\_COMMON\_\_WRITE\_ERROR\_MSG\_HPP

3

4 #include <ostream>

5 #include <stdexcept>

6

7 namespace stan {

8

9  namespace common {

10

11  void write\_error\_msg(std::ostream\* error\_stream,

12  const std::exception& e) {

13

14  if (!error\_stream) return;

15

16  \*error\_stream << std::endl

17  << "Informational Message: The current Metropolis proposal is about to be"

18  << " rejected because of the following issue:"

19  << std::endl

20  << e.what() << std::endl

21  << "If this warning occurs sporadically, such as for highly constrained"

22  << " variable types like covariance matrices, then the sampler is fine,"

23  << std::endl

24  << "but if this warning occurs often then your model may be either"

25  << " severely ill-conditioned or misspecified."

26  << std::endl;

27  }

28

29

30  } // namespace common

31

32 } // namespace stan

33

34 #endif

stan::common::write\_error\_msg

void write\_error\_msg(std::ostream \*error\_stream, const std::exception &e)

**Definition:** write\_error\_msg.hpp:11

stan::math::e

double e()

Return the base of the natural logarithm.

**Definition:** constants.hpp:86


---

[ Stan Home Page ]

*© 2011–2014,
Stan Development Team.*
